# Prevalence and factors associated with skin-to-skin contact (SSC) practice: findings from a population-based cross-sectional survey in 10 selected districts of Bangladesh

**DOI:** 10.1186/s12884-021-04189-3

**Published:** 2021-10-22

**Authors:** Nazia Binte Ali, Sabrina Sharmin Priyanka, Bal Ram Bhui, Samantha Herrera, Md. Rashidul Azad, Afsana Karim, Zubair Shams, Mahmoodur Rahman, S M Rokonuzzaman, Umme Salma Jahan Meena, Shams El Arifeen, Sk Masum Billah

**Affiliations:** 1 Maternal and Child Health Division, International Centre for Diarrhoeal Disease Research Bangladesh (icddr,b), Dhaka, Bangladesh; 2 Save the Children, Dhaka, Bangladesh; 3 PATH, Washington, DC USA; 4 The University of Sydney School of Public Health, Sydney, NSW 2006 Australia

**Keywords:** Skin to skin contact, Essential newborn care (ENC), Facility delivery, Caeserean section, Antenatal care (ANC), Sociodemographic Charcteristics, Low and middle income countries, Bangladesh

## Abstract

**Background:**

Skin-to-skin contact (SSC) practice improves newborn survival and child development through preventing hypothermia in newborns, improving early initiation of breastfeeding practice, and strengthening mother-child bonding. Despite having numerous benefits, it is one of the least practiced interventions in low and middle-income countries (1 to 74%). In Bangladesh, the prevalence of SSC was 26% in 2014. In this study, we aimed to estimate the prevalence of SSC in the study districts and identify factors that facilitate or inhibit SSC practice so that context-specific recommendations can be made to advance the use of this intervention.

**Methods:**

We used baseline household survey data of USAID’s MaMoni MNCSP project conducted in 10 districts of Bangladesh in 2019. Our analysis included 13,695 recently delivered women (RDW) with a live birth outcome. Our primary outcome was the mother’s reported practice of SSC. We examined various antepartum, intrapartum, newborn, and sociodemographic factors associated with SSC using a multivariable generalized linear model. Our findings were reported using adjusted Prevalence Risk Ratios (aPRRs) and 95% Confidence Intervals (CIs).

**Results:**

Overall, 28% of RDW reported practicing SSC across the 10 surveyed districts. Our multivariable analysis showed that public facility delivery (aPRR 2.01; 95%CI: 1.80, 2.26), private facility delivery (aPRR 1.23; 95%CI: 1.06, 1.42) and ≥ 4 antenatal care (ANC) visits at least one from a medically trained provider (MTP) (aPRR 1.17; 95%CI: 1.03, 1.26) had a significant positive association with SSC practice. Caesarean section (aPRR 0.64; 95%CI: 0.56, 0.73) had a significant negative association with SSC practice compared to vaginal births. We also found a significant positive association of SSC practice with mothers’ who perceived the birth size of their baby to be small, mothers with a higher education level (≥10 years), and mothers from households in the highest wealth quintile.

**Conclusions:**

The prevalence of SSC is very low in the surveyed districts of Bangladesh. Considering the factors associated with SSC, relevant stakeholders need to increase their efforts on improving ANC and facility delivery coverages as well as improving SSC practice in the facilities especially after caesarean deliveries. Countries with a high burden of home deliveries, also need to emphasize community-based interventions and increasing coverage of skilled birth attendance for improving this life-saving intervention.

## Background

The first 28 days of life, known as the neonatal period, is the most crucial time for newborn survival. Globally, around 2.5 million babies died in the neonatal period in 2017 [1]. Despite making significant improvements in the reduction of mortality among under-five children, the reduction of mortality in the neonatal period remains an unfinished Millennium Development Goal (MDG) agenda for many low and middle-income countries [2–5]. In Bangladesh, the neonatal mortality rate remains high at 30 per 1000 live births, constituting around 67% of all under-five deaths in 2017 [6]. To reduce newborn death, the World Health Organization (WHO) recommends a set of simple life-saving interventions, known as essential newborn care (ENC) practices [7, 8]. The core interventions included in ENC are early initiation of breastfeeding, thermal regulation (drying/wrapping the baby, skin-to-skin contact (SSC), and delayed bathing), and appropriate cord care [7, 8].. A significant positive association of SSC has been shown with thermal regulation and early initiation of breastfeeding practice [9].

WHO defines SSC as “*placing the baby naked on the mother’s bare chest, in a prone position covered by a cloth/blanket*” [10]. SSC is recommended to start immediately or within 10 min of vaginal births and as soon as the mother regains consciousness after cesarean section [11]. It is suggested to continue for an hour or longer if well tolerated by both the mother and the newborn [12]. The benefits of SSC on newborn and mother’s health are myriad. A recent Cochrane review of 46 randomized control trials including 3850 women and their infants suggested that immediate and early SSC (immediately after birth up till one hour) significantly improved the initiation of breastfeeding rates, duration of breastfeeding, and exclusive breastfeeding rates [13–15]. Newborns who received SSC had better stability in physiological parameters; cardiorespiratory parameters and blood glucose level [13]. Moreover, this critical period of contact provides an opportunity for neurobehavioral development of the newborn through the establishment of mother-infant attachment [13–15]. SSC benefits extend to the mother also, with improvements in the shortening of the third stage of labor, placental separation, uterine contraction, and milk let down [7].

Despite the numerous benefits, globally the more common practice is to separate the newborn from the mother after birth in both hospital and community settings [15]. A recent systematic review of literature from 28 countries found that the prevalence of SSC varied between 1 to 74% in the low and middle-income countries [16]. The review also showed a low prevalence of immediate SSC among countries in the South-East Asia region; with coverage in Nepal and India at only 15% [16]. Similar to other neighboring countries, the prevalence of SSC is low in Bangladesh at 26% in 2014 [7].

For Bangladesh to achieve the Sustainable Development Goal (SDG) target for neonatal mortality, at 12 deaths per 1000 live births by 2030, the country will need to substantially improve ENC coverage, especially SSC practice [17]. The first step in improving SSC practice is to identify the evidence on factors influencing the practice to develop context-specific solutions. The challenges in using existing evidence are that very few studies document the population-level determinants of SSC practice in Bangladesh, and the most recent data from Bangladesh on SSC practice are outdated (latest reflects data from 2012) and do not reflect the substantial changes in maternal care-seeking practices that have occurred over the past years [7]. For example, between 2014 and 2017, the coverage of four or more antenatal care (ANC) visits increased from 31 to 47%, while facility delivery increased from 37 to 50%. Both are important predictors of SSC [18]. Considering these evidence gaps, it is important to undertake an updated analysis using the most recent data to understand the current context of SSC practice in the local context.

In this study, we estimated the prevalence and determined the factors associated with SSC practice in 10 surveyed districts in Bangladesh. Findings from this study will generate updated evidence for program managers, researchers, and policymakers to develop and test context-specific solutions for improving coverage of this potentially life-saving intervention.

## Methods

### Data and settings

We used baseline household survey data of the MaMoni Maternal Newborn Care Strengthening Project (MNCSP) conducted in 10 districts in Bangladesh in 2019: Noakhali, Feni, Chandpur, Lakshmipur, Brahmanbaria, Habiganj, Manikganj, Faridpur, Madaripur, Khushtia. MaMoni MNCSP is a USAID-funded five-year project (2018–2022) focusing on strengthening maternal and newborn care interventions through the public health system in Bangladesh. This project aims to ensure equitable and high-quality maternal and newborn care service delivery, addressing health system challenges. Icddr,b, a member of the MaMoni MNCSP consortium, conducted the baseline population-based survey to measure coverage of key maternal and newborn health interventions.

The survey collected information from 14,982 recently delivered women (RDW) who had a birth outcome (abortion/stillbirth/live births) in the preceding 15 months using a structured questionnaire adapted from the Bangladesh Demographic Health Survey (BDHS) 2017 [18]. For this paper, we included 13,695 women who had a live birth outcome (1287 women having a stillbirth or abortion were excluded). We used data on women’s sociodemographic characteristics, newborn characteristics, ANC, place, and mode of delivery, and SSC practice, to estimate the prevalence and factors associated with SSC in the study areas.

### Sample size and sampling

The survey sample size was calculated to provide district-level estimates at a 5% level of precision for selected maternal, newborn, and family planning indicators. Considering the ±5% error margin and adjusting for clustering, an average of 120 clusters from each district with a cluster size of 10 was required. Our cluster size was 100 households. A household was defined as *“a person or group of related and unrelated persons who usually live together in the same dwelling unit(s), who have common cooking and eating arrangements, and who acknowledge one adult member as head of the household”* [19]. In this study, we included RDW with a birth outcome in the preceding 15 months to better evaluate changes over the life of the project. The sampling of clusters was done using the probability proportional to size (PPS) technique. The sampling frame for the PPS was prepared using census data from the Bangladesh Bureau of Statistics community series 2011 and population size for the study areas was adjusted using growth rates.

### Selection of study participants and data collection

In the selected cluster, data collectors (listers) first identified an index household using a random number table from the eligible couple register of the Family Welfare Assistants (FWA). The FWA are local resident community health workers who maintain a register of households in their community and provide maternal and family planning services. One hundred households were then listed starting from the index household in the clockwise direction to define the cluster boundary. The first listing included all RDW having any birth outcome (live birth, stillbirth, or abortion) in the three years preceding the survey to conduct a short survey to collect neonatal mortality data as it requires large sample size. Later, a second list of survey respondents (RDW with birth outcomes in the past 15 months of the survey) was generated from the first list prepared during the listing for collecting sociodemographic and careseeking practices. Interviewers approached all RDW with birth outcomes in the past 15 months and conducted full interviews in Bangla after obtaining informed written consent. Data collection was carried out between March and September 2019.

### Data quality assurance

To ensure the quality of data collection, our data collectors (listers/ interviewers) and field supervisors who had experience in survey data collection, were trained on the data collection tools between February and March 2019. Training included an in-depth review of the questionnaires, data collection methods, mock interviews, and field practice. Refresher training was provided as required. We used an Andriod-based system with a built-in consistency check system (range and skips) for minimizing data entry errors. A real-time dashboard was used for data quality monitoring. Supervisors and investigators conducted occasional field visits for observing data collection activities and provided post-visit feedback to the data collectors and supervisors in review meetings.

### Variable definitions

#### Outcome variables

Our primary outcome variable was reported SSC practice. We used mothers’ reported data for defining SSC [7]. Mothers were asked for their last live birth in the previous 15 months if “*the baby was placed naked directly on the bare skin of her chest immediately after birth.*” The responses were recorded as either yes or no.

#### Explanatory variables

We identified a list of explanatory variables based on an extensive literature review. Four domains of variables were tested; antepartum, intrapartum, newborn, and sociodemographic characteristics of the mother.

The frequency of ANC visits, at least one of which was from a medically trained provider (MTP), was considered as an antepartum factor. Categories include no ANC from an MTP, 1–3 ANC visits (at least one from an MTP), and ≥ 4 ANC visits (at least one from an MTP). Any ANC from a “*medical doctor, nurse, midwife, paramedic, family welfare visitor (FWV), community skilled birth attendant (CSBA), or medical assistant/sub-assistant community medical officer (MA/SACMO)*” was defined as ANC from an MTP.

Intrapartum factors included the places of delivery (home, public facility, private facility, and non-governmental organization [NGO] facilities), modes of delivery (vaginal births and cesarean sections), and whether the mother reported any complications during delivery. Any history of “*severe headache with blurry vision, convulsions, high blood pressure, excessive vaginal bleeding, prolonged labor>12 hours, retained placenta, and mal-presentation during delivery*” were included as complications during delivery.

We also examined newborn factors; including the mother’s perceived birth size of the baby (average, small or large), birth order (categories are 1–2, 3–4, and 5+), and sex of the baby.

The mother’s age categories, the highest level of educational attainment, current occupation (housewives, unskilled worker, and skilled worker), religion, and household wealth quintiles were the sociodemographic factors analyzed in this study. We used principal component analysis (PCA) for generating the household wealth index. Variables included in the PCA were housing characteristics, ownership of land, household assets, and household possession of domestic animals and transport vehicles. The PCA score was then divided into quintiles to assign household wealth quintile.

#### Data analysis

We first examined the frequency and distribution of all explanatory and outcome variables. Sample weight was calculated to adjust for the survey design. We reported numbers and weighted proportions for presenting descriptive statistics of our study population. In our bivariate analysis, we used generalized linear models (GLM) to examine the association of explanatory variables with SSC. Bivariate models were adjusted for weighting. Variables having a significant association in bivariate analysis were further examined using a multivariable model. GLM was also used for multivariable analysis. We specified binomial family, log link function (as our outcome is binary), robust estimator (to adjust for nonparametric distribution), and adjusted for sampling weight in our multivariable model. Findings from the bivariate and multivariable models were reported using prevalence rate ratios (PRRs) and 95% confidence intervals. We consider a *p*-value < 0.05 as significant. Stata 14 statistical software was used for analysis.

#### Ethics

The survey received ethical approval from the Institutional Review Board of icddr,b and Save the Children US Ethical Review Committee. The survey was conducted following ethical principles of research with human participants. Data collectors read out the informed consent forms and explained the purpose of the survey, risks, benefits, use of data, procedures for ensuring privacy and confidentiality, and the voluntary nature of participation to the study participants. Data collection commenced upon receiving informed written consent.

## Results

Out of 13,695 mothers included in the study, more than half (60%) were between the age of 20 to 29 years (Table 1). Almost two-thirds of mothers had some secondary or higher education while only 7% of mothers had no formal education. The majority of mothers surveyed were housewives (97%).

**Table 1:** Sociodemographic, antepartum, intrapartum, and newborn characteristics of RDWs in surveyed districts of Bangladesh, 2019

Variables	Categories	Frequency (n) ***N*** = 13,695	Percentage^a^ % (95%CI)
Sociodemographic characteristics			
Mother’s age	15–19	2028	14.8 (14.2, 15.4)
	20–29	8166	59.6 (58.8, 60.4)
	30–39	3318	24.2 (23.5, 24.9)
	40–49	183	1.3 (1.1, 1.5)
Mother’s educational level	No formal education	986	7.2 (6.8, 7.6)
	Primary incomplete (1–4 years)	1570	11.5 (10.9, 12.0)
	Primary complete (5 years)	2024	14.8 (14.2, 15.4)
	Secondary incomplete (6–9 years)	6205	45.3 (44.5, 46.1)
	Secondary and above (≥10 years)	2910	21.3 (20.6, 21.9)
Mother’s current occupation	Housewives	13,268	96.9 (96.6, 97.2)
	Skilled worker	375	2.7 (2.5, 3.0)
	Unskilled worker	52	0.4 (0.3, 0.5)
Mother’s religion	Muslim	12,912	94.3 (93.9, 94.7)
	Hindu /others	783	5.7 (5.3, 6.1)
Wealth Quintile	Lowest	2657	19.4 (18.7, 20.1)
	Second	2671	19.5 (18.8, 20.2)
	Middle	2777	20.3 (19.6, 20.9)
	Fourth	2758	20.1 (19.5, 20.8)
	Highest	2832	20.7 (20.0, 21.4)
Antepartum characteristics			
Frequency of ANC visits (at least one from a medically trained provider (MTP))	No ANC from MTP	3948	28.8 (28.0, 29.6)
	1–3 ANC (at least one from an MTP)	6091	44.5 (43.6, 45.3)
	≥4 (at least one from an MTP)	3656	26.7 (25.9, 27.4)
Intrapartum characteristics			
Places of delivery	Home delivery	6417	46.9 (46.0, 47.7)
	Public facility	2040	14.9 (14.3, 15.5)
	Private facility	5025	36.7 (35.9, 37.5)
	NGO facility	133	0.9 (0.8, 1.1)
	Missing	80	0.6 (0.4, 0.7)
Mode of delivery	Vaginal birth	8774	64.1 (63.2, 64.9)
	Caeserean section	4914	35.9 (35.1,36.7)
	Missing	9	0.1 (0.0,0.1)
Any reported complication during delivery	No	10,743	78.4 (77.7, 79.1)
	Yes	2952	21.5 (20.9, 22.2)
Newborn characteristics			
Birth size	Small	1363	9.9 (9.5, 10.5)
	Average	9393	68.6 (67.8, 69.4)
	Large	2893	21.1 (20.4, 21.8)
	Missing	46	0.3 (0.2, 0.4)
Birth order	1–2	8787	64.2 (63.4, 64.9)
	3–4	4149	30.3 (29.5, 31.1)
	5+	759	5.5 (5.2, 5.9)
Sex of the child	Male	7017	51.2 (50.4, 52.1)
	Female	6609	48.3 (47.4, 49.1)
	Missing	69	0.5 (0.3, 0.6)

^a^weighted

**Fig. 1:**
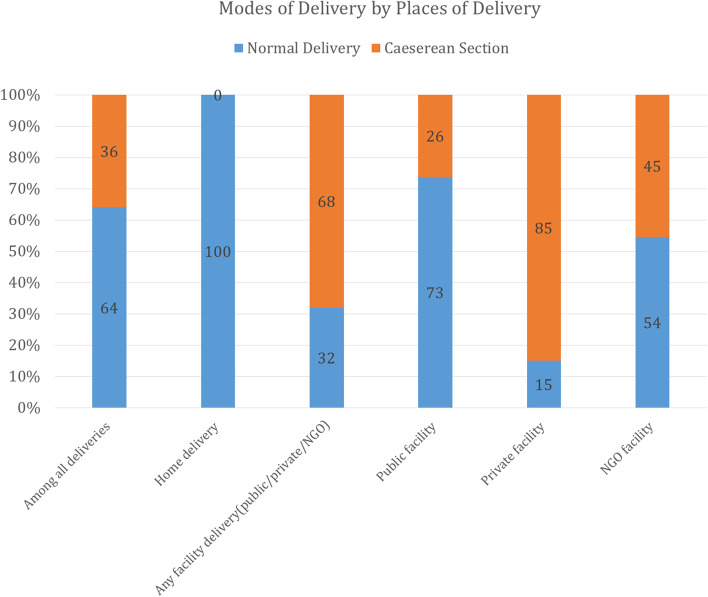
Distribution of mode of delivery by place of delivery in surveyed districts of Bangladesh, 2019*

Overall, 29% of the study participants did not report any ANC from an MTP whereas 45% reported 1–3 ANC (at least one from an MTP) and 27% reported receiving ≥ 4 ANC (at least one from an MTP) (Table 1). Forty-seven percent of the mothers delivered at home, 37% in a private facility, and 15% in a public facility. Among all surveyed mothers, the prevalence of cesarean section was high at 36%. The majority of the mothers who delivered in the private facilities reported cesarean deliveries (85%) while less than one-third of mothers who delivered in the public facilities reported having a cesarean delivery (Fig. 1). One in five mothers reported experiencing complications during delivery. More than two-thirds of mothers reported that the perceived size of their child at birth was average while one in 10 mothers reported that their child was small at birth. Around 64% of the babies were first or second born.

### Prevalence of reported SSC practice

**Fig. 2:**
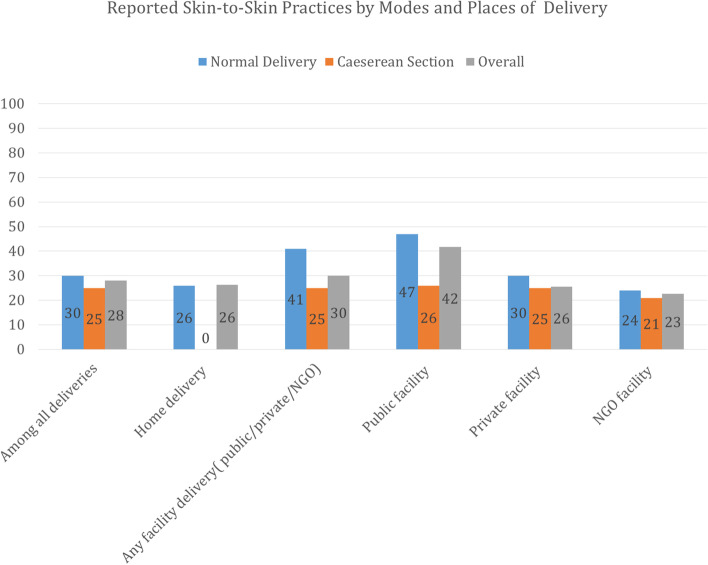
Distribution of SSC by mode and place of delivery in surveyed districts of Bangladesh, 2019

Overall, 28% of mothers reported SSC practice (Table 2). The reported practice of SSC was higher among mothers who had ≥ 4 ANC visits (at least one from an MTP) (30%; 95% CI: 28.8, 31.8) compared to mothers who had no ANC from an MTP (25%; 95% CI: 23.9, 26.7). More mothers who delivered in the public facilities reported practicing SSC (42%; 95% CI: 39.5, 43.8) with their newborn than mothers who delivered at home (26%; 95% CI: 25.3, 27.5) or in a private facility (26%; 95% CI: 24.3, 26.7). The practice of SSC was lower among mothers who delivered by cesarean section (25%; 95% CI: 23.8, 26.2) compared to those who had a vaginal birth (30%; 95% CI: 29.2, 31.2). Figure 2 showed that reported SSC practice were higher for vaginal births in both public facilities and private facilities compared to home deliveries, however, the opposite was observed for cesarean deliveries.

**Table 2:** SSC prevalence by antepartum, intrapartum, newborn, and sociodemographic factors in surveyed districts of Bangladesh, 2019

Variables	Categories	Overall (***N*** = 13,695)	Frequency (***n*** = 3878)	Percentages^a^ % (95% CI)
The overall prevalence of SSC		**13,695**	**3878**	**28.3 (27.6, 29.1)**
Antepartum characteristics				
Frequency of antenatal care(ANC) visits with at least one ANC from a medically trained provider (MTP))	No ANC from MTP	3948	1000	25.3 (23.9, 26.7)
	1–3 ANC (at least 1 from an MTP)	6091	1772	29.1 (27.9, 30.2)
	≥4 (at least 1 from an MTP)	3656	1107	30.3 (28.8, 31.8)
Intrapartum characteristics				
Place of delivery	Home delivery	6417	1694	26.4 (25.3, 27.5)
	Public facility	2040	850	41.7 (39.5, 43.8)
	Private facility	5025	1282	25.5 (24.3, 26.7)
	NGO facility	133	30	22.6 (15.7, 30.0)
Mode of deliveries	Vaginal birth	8774	2650	30.2 (29.2, 31.2)
	Caeserean section	4914	1227	25.0 (23.8,26.2)
Any complication during delivery	No	10,743	2962	27.6 (26.7, 28.4)
	Yes	2952	917	31.1 (29.4, 32.8)
Newborn characteristics				
Birth size	Small	1363	464	34.0 (31.5, 36.6)
	Average	9393	2637	28.1 (27.2, 28.9)
	Large	2893	770	26.6 (25.0, 28.3)
Birth order	1–2	8787	2548	29.0 (28.1, 29.9)
	3–4	4149	1150	27.7 (26.4, 29.1)
	5+	759	181	23.8 (20.8, 27.0)
Sex of the child	Male	7017	2019	28.8 (27.7, 29.8)
	Female	6609	1838	27.8 (26.7, 28.9)
Sociodemographic characteristics				
Mother’s age	15–19	2028	591	29.1 (28.3, 32.5)
	20–29	8166	2324	28.5 (28.6, 30.6)
	30–39	3318	926	27.9 (28.6,30.6)
	40–49	183	38	20.9 (15.8, 28.6)
Mother’s educational level	No formal education	986	243	24.6 (21.9, 27.5)
	Primary incomplete (1–4 years)	1570	379	24.2 (22.1, 26.4)
	Primary complete (5 years)	2024	504	24.9 (23.0, 26.8)
	Secondary incomplete (6–9 years)	6205	1829	29.5 (28.3, 30.6)
	Secondary and above (≥10 years)	2910	923	31.7 (30.0, 33.4)
Mother’s current occupation	Housewives	13,268	3773	28.4 (27.7, 29.2)
	Skilled worker	375	92	24.7 (20.3, 29.2)
	Unskilled worker	52	14	26.6 (15.6, 41.0)
Mother’s religion	Muslim	12,912	3642	28.2 (27.4, 28.9)
	Hindu /others	783	237	30.2 (27.1, 33.6)
Wealth quintile	Lowest	2657	664	25.0 (23.3, 26.7)
	Second	2671	706	26.4 (24.8, 28.2)
	Middle	2777	813	29.3 (27.6, 31.0)
	Fourth	2758	827	29.9 (28.3, 31.7)
	Highest	2832	868	30.7 (30.0, 32.4)

^a^ Weighted

Among the newborn characteristics, the reported practice of SSC decreased with increasing perceived birth size of the baby. SSC was reported among 34% (95% CI: 31.5, 36.6) of babies perceived to be small, 28% (95% CI: 27.2, 28.9) of babies perceived to be average, and 27% (95% CI: 24.9, 28.2) of babies perceived to be large. The practice of reported SSC declined with increasing birth order; 29% (95% CI: 28.1, 29.9) among first or second birth order, 28% (95% CI: 26.4, 29.1) among third or fourth birth order, and 24% (95% CI: 20.8, 27.0) among fifth or higher birth order. Mothers with a higher level of education and those from households in the higher wealth quintiles reported a higher prevalence of SSC.

### Factors associated with SSC practice

Table 3 illustrates the adjusted and unadjusted association of SSC with antepartum, intrapartum, newborn, and sociodemographic factors. Our bivariate analysis shows a significant positive association of SSC with 1–3 ANC visits at least one from an MTP, four or more ANC visits at least one of which is from an MTP during pregnancy, delivery in a public facility, the experience of complications during delivery, perceived smaller newborn birth size, mother’s secondary level of educational attainment, and household wealth quintile. On the other hand, having a cesarean section, increasing birth order, and mother’s age between 40 and 49 years, were inversely associated with SSC.

**Table 3:** Factors associated with SSC in surveyed districts of Bangladesh, 2019

		Bivariate analysis		Multivariable analysis	
Variables	Categories	uPRR^**1**^	95% CI	aPRR^**2**^	95% CI
Antepartum characteristics					
Frequency of antenatal care (ANC) visits with at least one ANC from a medically trained provider (MTP)	No ANC from an MTP	ref		ref	
	1–3 ANC (at least 1 from an MTP)	1.16***	1.09, 1.24	1.14**	1.03, 1.26
	≥4 (at least 1 from an MTP)	1.23***	1.14, 1.32	1.17*	1.04, 1.30
Intrapartum characteristics					
Place of delivery	Home delivery	ref		ref	
	Public facility	1.60***	1.50, 1.71	2.01***	1.80, 2.26
	Private facility	1.02	0.95, 1.09	1.23**	1.06, 1.42
	NGO facility	0.85	0.62, 1.17	0.88	0.58, 1.33
Mode of delivery	Vaginal birth	ref		ref	
	Caesarean section	0.88***	0.83, 0.93	0.64***	0.56, 0.73
Any complication during delivery	No	ref		ref	
	Yes	1.13***	1.06, 1.20	1.13**	1.04, 1.25
Newborn characteristics					
Birth size	Average	ref		ref	
	Small	1.21*	1.13, 1.32	1.28***	1.12, 1.45
	Large	0.95	0.89, 1.02	0.95	0.86, 1.04
Birth order	1–2	ref		ref	
	3–4	0.95	0.90, 1.01	0.97	0.88, 1.07
	5+	0.80*	0.70, 0.92	0.86	0.70, 1.07
Sex of the child	Female	ref			
	Male	1.04	0.98, 1.10		
Sociodemographic characteristics					
Mother’s age	15–19	ref		ref	
	20–29	0.97	0.90, 1.05	1.00	0.90, 1.13
	30–39	0.96	0.88, 1.05	1.08	0.92, 1.25
	40–49	0.71*	0.53, 0.96	0.83	0.55, 1.23
Mother’s educational level	No formal education	ref		ref	
	Primary incomplete (1–4 years)	0.98	0.84, 1.12	0.95	0.78, 1.15
	Primary complete (5 years)	1.01	0.88, 1.16	0.97	0.80, 1.16
	Secondary incomplete (6–9 years)	1.20*	1.07, 1.35	1.18*	1.00, 1.41
	Secondary and above (≥10 years)	1.31***	1.16, 1.48	1.31**	1.09, 1.60
Mother’s current occupation	Housewives	ref			
	Skilled worker	0.89	0.74, 1.06		
	Unskilled worker	0.91	0.57, 1.47		
Mother’s religion	Muslim	ref			
	Hindu /others	1.09	1.00, 1.21		
Wealth Quintile	Lowest	ref		ref	
	Second	1.06	1.01, 1.17	1.01	0.89, 1.15
	Middle	1.18***	1.08, 1.29	1.14*	1.00, 1.29
	Fourth	1.21***	1.10, 1.32	1.14	1.00, 1.30
	Highest	1.25***	1.14, 1.36	1.17*	1.02, 1.35

* Pvalue< 0.05

* *Pvalue< 0.001

* **Pvalue< 0.000

^1^Unadjusted Prevalence Rate Ratios

^2^Adjusted Prevalence Rate Ratios

Our multivariable model found a significant positive association of SSC with receving one to three ANC visits (at least one from an MTP) (aPRR 1.14; 95% CI: 1.03, 1.26) and ≥ 4 ANC visits (at least one from an MTP) (aPRR 1.17; 95% CI: 1.03, 1.26) compared to mothers who reported no ANC from an MTP. Compared to the women who delivered at home, delivery at a health facility increased SSC practice by two-fold for public facilities (aPRR 2.01; 95% CI: 1.80, 2.26) and 23% for private facilities (aPRR 1.23; 95% CI: 1.06, 1.42). Women who had a caesarean section were significantly less likely to practice SSC; (aPRR 0.64; 95% CI: 0.56, 0.73) compared to women who had a vaginal birth. Newborns with mothers having complications during delivery were more likely to receive SSC than their counterparts. Babies who were perceived to be small at birth had a higher likelihood of receiving SSC compared to babies perceived to be average size; (aPRR 1.28; 95% CI: 1.12, 1.45). Mothers having higher educational attainment were more likely to report SSC compared to women having no formal education. Newborns of mothers from households in the highest wealth quintile had a significantly higher likelihood of receiving SSC (aPRR 1.17; 95% CI: 1.02, 1.35) than the newborns of mothers from households in the lowest wealth quintile.

## Discussion

Skin-to-skin contact is a key ENC interventions that improve newborn survival [9, 20]. The prevalence of reported SSC practice was low at 28% in the ten surveyed districts in Bangladesh. Results from our multivariable model identified two critical positive factors for improving SSC practice: i) facility delivery and ii) ANC from MTPs. In contrast, cesarean delivery significantly reduces the practice of SSC in facilities. Researchers, program implementers, and policymakers need to consider these determinants when identifying and designing essential interventions to improve SSC practice at the population level.

Despite being a simple and no-cost intervention with substantial health benefits, SSC is one of the least practiced ENC interventions in developing countries [15, 16, 21, 22]. Our findings reiterate this evidence for 10 surveyed districts in Bangladesh. A similarly low prevalence of SSC practice was reported in previous studies in Bangladesh, which showed a prevalence between 26 and 30% [7, 23]. Our reported prevalence of SSC is higher than the reported prevalence in India (14.5%), Nepal (16.5%), Tanzania (9.7%), and Ethiopia (24.3%). It is lower than the reported prevalence in Sri Lanka at 50% and the Gambia at 35.7% [23–27]. The low prevalence of SSC practice in these developing countries could be attributed to various factors such as the high burden of home deliveries, low ANC coverage and low availability of community-based interventions, and sociodemographic and health system factors [23, 24, 26–28].

One striking finding of our study is that mothers who delivered in the facilities have a higher likelihood of SSC practice compared to the mothers who delivered at home. Previous studies from Bangladesh, Gambia, and Ethiopia also found facility delivery to be a significant positive predictor of SSC practice [7, 26, 27]. Although Bangladesh is observing an increasing trend in coverage of facility deliveries, 50% of women are still delivering at home [6]. This finding highlights the need for increasing coverage of facility delivery as well as improving SSC practice after home deliveries in Bangladesh. Lack of awareness, social norms, distance to the facilities, lack of transport facilities, cost of care-seeking, and poor quality of care are some of the key barriers preventing women from delivering in facilities in rural areas of Bangladesh [29–34]. The Alliance for Maternal and Newborn Health Improvement (AMANHI) study which implemented 24/7 obstetric services, training for health care providers, community advocacy, comprehensive birth planning counseling, financial incentives for covering delivery costs, and emergency referral transport in Sylhet Division of Bangladesh, demonstrated significantly improved coverage of facility deliveries from 25 to 78% in the intervention areas [35]. Policymakers and health programmers need to consider a similar integrated package of interventions addressing both supply-side and demand-side barriers to improve coverage of facility deliveries in this setting.

On the other hand, to improve SSC practice after home deliveries, community-based interventions such as community skilled birth attendants (CSBA) may play a significant role. A community-based maternal and newborn health package that was piloted in Bangladesh, Malawi, Nepal, and India showed significant improvement in all four ENC practices, including SSC [24, 28]. The study deployed trained community health workers/volunteers for regular home visits during and soon after delivery. Community health workers/volunteers provided routine maternal and newborn care, promoted routine health service utilization, supported birth preparedness, conducted counseling on danger signs and ENC, and identified and referred women and newborns having maternal and newborn danger signs to the nearest health facilities during regular home visits. Although community-based interventions showed some positive impact on ENC practices, considering very low coverage of SBA among home deliveries (3%) in Bangladesh and resource-intensive nature of the community based maternal and newborn health programming, the question remains “*which investment will bring maximum impact on SSC practice in these resource-limited settings; facility delivery or CSBA or both?*” [18, 23].

Another important finding of our study is that despite having a significant positive association of facility delivery on SSC practice, less than half of the women who delivered in the facilities reported SSC. This signifies that increasing coverage of facility delivery alone will not be able to bring the change in SSC practice without improving its practice in the facilities. Shortage of a skilled workforce, lack of time, lack of step by step protocol for initiation of SSC, interference with routine procedures after birth, lack of motivation among the mothers and family members, and cultural practices were identified as some of the major barriers in implementing SSC after facility deliveries [36–38]. WHO and UNICEF recommend implementation of the baby-friendly hospital initiatives to improve SSC and breastfeeding practices through training of the health care providers and creating an enabling hospital environment for SSC, for example by providing standard guidelines on SSC, ensuring availability of a skilled workforce and sufficient postnatal beds with the provision of privacy [12, 39]. The Government of Bangladesh has already started the implementation of baby-friendly hospital initiatives in public health facilities. Experience from implementation showed a significant positive impact on improving SSC and early initiation of breastfeeding practices in several other settings [40, 41]. Additionally, strengthening monitoring and adoption of essential measures to address the barriers and challenges of implementing SSC in the facilities using the Plan-Do-Act-Check (PDCA) model may improve SSC practice in facilities [42–44]. Save the Children’s MaMoni MNCSP project has recently started implementing the PDCA model for improving ENC practices, including SSC, in the public health facilities of Manikganj and Madaripur district of Bangladesh. Evaluation of this PDCA model will generate evidence on the effectiveness of this intervention in improving SSC practice in the local contexts.

Our results showed that having a cesarean delivery reduced the likelihood of receiving SSC by 36% compared to the mothers having a vaginal birth. Findings from other studies also suggested a negative impact of cesarean section on SSC practice and early initiation of breastfeeding [11, 45–49]. WHO recommends starting SSC immediately (if possible in the operation theater) after cesarean delivery as soon as the mother regains consciousness in the absence of any precarious complications [12]. For uncomplicated cesarean deliveries performed using spinal anesthesia, SSC can be initiated immediately in the operation theater. Two different intervention modalities, such as the Plan, Do Study, Act (PDSA) model and Practice Reflection, Education, and training, Combined with the Ethnography for Sustainable Success (PRECESS) model have shown a significant improvement in SSC practice after cesarean births [50–53]. The PDSA model piloted SSC in the operation theater using a flow chart and developed a monitoring and feedback mechanism to address the barriers and challenges of implementation. The PRECESS project educated the staff on standard steps of SSC implementation, created a staff monitoring mechanism on SSC after cesarean births, and shared videos with mothers on SSC [50, 51]. One limitation of these studies is that the sample sizes are small. Contextual adaptation of these evidence-based interventions must be tested using a rigorous implementation research design, with a sufficient sample size for generating strong evidence on the efficacy of these interventions in the local context.

Another challenge in improving SSC practice in Bangladesh is the low coverage of SSC in private sector and NGO facilities. High rates of cesarean deliveries in private facilities (85%) and NGO facilities (45%) could be a reason for such low coverage of SSC given our results showed cesarean deliveries to be a negative predictor of SSC .. National-level data from Bangladesh have shownthat 84% of women who delivered in a private facility had a cesarean section [18]. The WHO threshold of optimal cesarean section rates for any country is 15% which signifies that unnecessary cesarean deliveries are happening mostly in the profit-driven private sectors [54]. It’s crucial to implement proven interventions such as accreditation of private facilities, implementation of Robson’s classification, and the second review to regulate unnecessary cesarean sections in the private facilities [55–57]. In essence, implementing programs to initiate SSC after cesarean sections in the operating theater along with optimization of cesarean deliveries are necessary to bring about the potential improvement in SSC practice.

Our study also showed that mothers having one to three ANC visits and four or more ANC visits (at least one from an MTP) had better reported SSC practice. Our finding is consistent with previous studies in Bangladesh, Gambia, and Ethiopia [7, 26, 27]. ANC has the potential to improve SSC practice in two ways. First, ANC is an important predictor of facility delivery and it is evident from our findings that facility delivery improves the likelihood of SSC practice [58–61]. Second, lack of motivation among the mother and the family members is a potential barrier to SSC practice [36, 38]. Recent evidence identifies ANC counseling as a potential opportunity for motivating mothers for uptake of SSC [36].

This study has the following limitations. First, we used mothers’ self-reported data for defining SSC practice. Previous studies reported mixed evidence on the validity of using mothers’ reported data for defining SSC compared to observation-based data [62, 63]. However, observation-based data collection is expensive and not feasible for population-based surveys. Additionally, we found that major national-level population-based surveys such as the Demographic Health Survey in Bangladesh and Nigeria collect mothers’ reported data for defining SSC and we adopted questions from BDHS for defining SSC in this study [7]. We recommend further exploration to identify appropriate wording for defining SSC during population-level surveys and to collect more in-depth information regarding the actual timing of the start and duration of SSC to improve validity. Second, recall bias may also be an issue which we tried to address by interviewing women with shorter recall periods in this survey [18].

Despite the limitations, our study is unique in terms of generating evidence on the prevalence and factors associated with SSC practice, which are not often studied at a population level [7]. It is crucial to monitor the population-level estimates of SSC and to understand the factors influencing its practice, to better understand the barriers to its uptake, and what can be done to improve coverage of the practice in the local context.

## Conclusions

There is no doubt that SSC has numerous benefits for both the mother and the baby [64]. It is high time to act to improve SSC practice in the facilities and coverage of facility deliveries. Implementation of the baby-friendly hospital initiative may improve the practice among the health care providers and create an enabling environment to increase SSC practice after facility deliveries. Emphasis is needed on the initiation of SSC after the cesarean section in the absence of any precarious maternal and newborn complication. It is also important to regulate unnecessary cesarean deliveries. To increase facility deliveries, it is important to address both the supply and demand-side barriers through an integrated package of interventions. Community-based interventions are important for improving coverage of SSC practice, especially in settings with a high burden of home births. ANC from MTPs could be the potential platform for not only encouraging mothers for facility deliveries but also counseling mothers on this potentially lifesaving intervention. Further research is needed to test evidence-based solutions for improving SSC practice in the local context.

## Data Availability

Due to ethical restrictions related to protecting study participants privacy and confidentiality, data access is restricted by the Ethical Review Committee of icddr,b. According to the icddr,b data policy (http://www.icddrb.org/policies), interested parties may contact Ms. Armana Ahmed (aahmed@icddrb.org) with further inquiries related to data access.

## Abbreviations

ANC: Antenatal Care
BDHS: Bangladesh Demographic and Health Survey
CSBA: Community Skilled Birth Attendent
DHS: Demographic and Health Survey
ENC: Essential Newborn Care
GLM: Generalized Linear Model
MaMoni MNCSP: MaMoni Maternal Newborn Care Strengthening Project
MTP: Medically Trained Provider
NGO: Non Government Organization
PDCA: Plan DO Check Act
PDSA: Plan, Do Study, Act
PRECESS: Practice Reflection, Education, and training, Combined with the Ethnography for Sustainable Success
RDW: Recently Delivered Women
SSC: Skin-to-Skin Contact
UNICEF: United Nations Children’s Fund
USAID: United States Agency for International Development
WHO: World Health Organization

## References

[1] Hug L, Alexander M, You D, Alkema L (2019). Estimation UNI-aGfCM. National, regional, and global levels and trends in neonatal mortality between 1990 and 2017, with scenario-based projections to 2030: a systematic analysis. Lancet Glob Health.

[2] WHO. Global Health Observatory (GHO) data 1990, 2017. Available from: https://www.who.int/gho/child_health/mortality/neonatal_infant_text/en/.

[3] WHO. Success factors for women’s and children’s health: Rwanda. 2015.

[4] Unicef. Millenium Development Goals. 2014. Available from https://data.unicef.org/resources/the-millennium-development-goals-report-2014/.

[5] Victora CG, Requejo JH, Barros AJ, Berman P, Bhutta Z, Boerma T (2016). Countdown to 2015: a decade of tracking progress for maternal, newborn, and child survival. Lancet (London, England).

[6] National Institute of Population Research and Training (NIPORT), Mitra and Associates (MA), International I (2019). Bangladesh Demographic and Health Survey 2017.

[7] Singh K, Khan SM, Carvajal-Aguirre L, Brodish P, Amouzou A, Moran A (2017). The importance of skin–to–skin contact for early initiation of breastfeeding in Nigeria and Bangladesh. J Glob Health.

[8] Darmstadt GL, Kinney MV, Chopra M, Cousens S, Kak L, Paul VK (2014). Who has been caring for the baby?. Lancet.

[9] Khadivzadeh T, Karimi A (2009). The effects of post-birth mother-infant skin to skin contact on first breastfeeding.

[10] Cadwell K, Brimdyr K, Phillips R (2018). Mapping, measuring, and analyzing the process of skin-to-skin contact and early breastfeeding in the first hour after birth. Breastfeed Med.

[11] Stevens J, Schmied V, Burns E, Dahlen H (2014). Immediate or early skin-to-skin contact after a caesarean section: a review of the literature. Matern Child Nutr.

[12] WHO. Protecting, Promoting and Supporting Breastfeeding in Facilities Providing Maternity and Newborn Services. 2017. Available from: https://apps.who.int/iris/handle/10665/259386. 29565522

[13] Moore ER, Anderson GC, Bergman N, Dowswell T (2012). Early skin-to-skin contact for mothers and their healthy newborn infants. Cochrane Database Syst Rev.

[14] Moore ER, Anderson GC, Bergman N. Early skin-to-skin contact for mothers and their healthy newborn infants. Cochrane Database Syst Rev. 2007;(3):CD003519. 10.1002/14651858.CD003519.pub2. Update in: Cochrane Database Syst Rev. 2012;5:CD003519. PMID: 17636727. 10.1002/14651858.CD003519.pub3 PMC3979156 22592691

[15] Moore ER, Bergman N, Anderson GC, Medley N (2016). Early skin-to-skin contact for mothers and their healthy newborn infants. Cochrane Database Syst Rev.

[16] Abdulghani N, Edvardsson K, Amir LH (2018). Worldwide prevalence of mother-infant skin-to-skin contact after vaginal birth: A systematic review. PLoS One.

[17] UN. Suatainable Development Goals: United Nations; 2015. Available from: http://www.un.org/sustainabledevelopment/health/.

[18] National Institute of Population Research and Training (NIPORT), and ICF. Bangladesh Demographic and Health Survey 2017-18: Key Indicators. Dhaka, Bangladesh, and Rockville, Maryland, USA: NIPORT, and ICF; 2019.

[19] National Institute of Population Research and Training (NIPORT), Mitra and Associates, and ICF International. 2016. Bangladesh Demographic and Health Survey 2014. Dhaka, Bangladesh, and Rockville, Maryland, USA: NIPORT, Mitra and Associates, and ICF International. National Institute of Population Research and Training (NIPORT). Bangladesh Demographic and Health Survey. The DHS Program, Ministry of Health and Family Welfare GoB; 2014 March 2016. Report No: FR311.

[20] Barros FC, Bhutta ZA, Batra M, Hansen TN, Victora CG, Rubens CE (2010). Global report on preterm birth and stillbirth (3 of 7): evidence for effectiveness of interventions. BMC Pregnancy Childbirth.

[21] de Graft-Johnson J, Vesel L, Rosen HE, Rawlins B, Abwao S, Mazia G (2017). Cross-sectional observational assessment of quality of newborn care immediately after birth in health facilities across six sub-Saharan African countries. BMJ Open.

[22] de Jonge E, Azad K, Hossen M, Kuddus A, Manandhar DS, van de Poel E (2018). Socioeconomic inequalities in newborn care during facility and home deliveries: a cross sectional analysis of data from demographic surveillance sites in rural Bangladesh, India and Nepal. Int J Equity Health.

[23] Crowe S, Prost A, Hossen M, Azad K, Kuddus A, Roy S, Nair N, Tripathy P, Saville N, Sen A, Sikorski C. Generating insights from trends in newborn care practices from prospective population-based studies: examples from India, Bangladesh and Nepal. PloS one. 2015;10(7):e0127893. 10.1371/journal.pone.0127893 PMC4503724 26176535

[24] Upadhyay RP, Rai SK, Anand KJAP (2012). Community neonatal practice and its association with skilled birth attendance in rural Haryana. India..

[25] Cederfeldt J, Carlsson J, Begley C, Berg M. Quality of intra-partum care at a university hospital in Nepal: A prospective cross-sectional survey. Sex Reprod Healthc. 2016;7:52–7. 10.1016/j.srhc.2015.11.004 26826046

[26] Nigatu D, Abeje G, Mekonnen AG, Azage M, Bogale D (2020). Maternal Health Service Uptake Is Associated with a Higher Skin-to-Skin Care Practice in Ethiopia: Result from a National Survey. Biomed Res Int.

[27] Ekholuenetale M, Onikan A, Ekholuenetale CE. Prevalence and determinants of mother and newborn skin-to-skin contact in The Gambia: a secondary data analysis. J Egypt Public Health Assoc. 2020;95(1):1–9. 10.1186/s42506-020-00050-1 PMC7423813 32813211

[28] Sitrin D, Guenther T, Waiswa P, Namutamba S, Namazzi G, Sharma S (2015). Improving newborn care practice through home visits: lessons from Malawi, Nepal, Bangladesh, and Uganda. Glob Health Action.

[29] Bohren MA, Hunter EC, Munthe-Kaas HM, Souza JP, Vogel JP, Gülmezoglu AM (2014). Facilitators and barriers to facility-based delivery in low- and middle-income countries: a qualitative evidence synthesis. Reprod Health.

[30] Borghi J, Ensor T, Somanathan A, Lissner C, Mills A (2006). Mobilising financial resources for maternal health. Lancet (London, England).

[31] Nahar S, Costello A (1998). The hidden cost of 'free' maternity care in Dhaka, Bangladesh. Health Policy Plan.

[32] Nguyen HT, Hatt L, Islam M, Sloan NL, Chowdhury J, Schmidt JO (2012). Encouraging maternal health service utilization: an evaluation of the Bangladesh voucher program. Soc Sci Med (1982).

[33] Saksena P, Xu K, Elovainio R, Perrot J (2012). Utilization and expenditure at public and private facilities in 39 low-income countries. Trop Med Int Health.

[34] Sarker BK, Rahman M, Rahman T, Hossain J, Reichenbach L, Mitra DK (2016). Reasons for preference of home delivery with traditional birth attendants (TBAs) in rural Bangladesh: a qualitative exploration. PLoS One.

[35] Rahman S, Choudhury AA, Khanam R, Moin SMI, Ahmed S, Begum N (2017). Effect of a package of integrated demand- and supply-side interventions on facility delivery rates in rural Bangladesh: Implications for large-scale programs. PLoS One.

[36] Alenchery AJ, Thoppil J, Britto CD, de Onis JV, Fernandez L, Suman Rao PN (2018). Barriers and enablers to skin-to-skin contact at birth in healthy neonates - a qualitative study. BMC Pediatr.

[37] Chan G, Bergelson I, Smith ER, Skotnes T, Wall S (2017). Barriers and enablers of kangaroo mother care implementation from a health systems perspective: a systematic review. Health Policy Plan.

[38] Abdulghani N, Edvardsson K, Amir LH (2020). Health care providers' perception of facilitators and barriers for the practice of skin-to-skin contact in Saudi Arabia: a qualitative study. Midwifery..

[39] WHO (2009). Baby-friendly hospital initiative: revised, updated and expanded for integrated care.

[40] Philipp BL (2017). The Importance of the Baby-Friendly Hospital Initiative. JAMA Pediatr.

[41] Rowe-Murray HJ, Fisher JR (2002). Baby friendly hospital practice: cesarean section is a persistent barrier to early initiation of breastfeeding. Birth..

[42] Saxena S, Ramer L, Shulman IA (2004). A comprehensive assessment program to improve blood-administering practice using the FOCUS–PDCA model. Transfusion..

[43] Redick EL (1999). Applying FOCUS-PDCA to solve clinical problems. Dimensions Crit Care Nurs.

[44] Farahbakhsh M, Tabrizi J, NIKNIAZ A (2010). The use of Focus-PDCA in primary health care performance improvement: case study of East Azerbaijan health centers.

[45] Brady K, Bulpitt D, Chiarelli C (2014). An interprofessional quality improvement project to implement maternal/infant skin-to-skin contact during cesarean delivery. J Obstet Gynecol Neonatal Nurs.

[46] Wu Y, Wang Y, Huang J, Zhang Z, Wang J, Zhou L (2018). The association between caesarean delivery and the initiation and duration of breastfeeding: a prospective cohort study in China. Eur J Clin Nutr.

[47] Karim F, Billah SM, Chowdhury MA, Zaka N, Manu A, Arifeen SE, Khan AN. Initiation of breastfeeding within one hour of birth and its determinants among normal vaginal deliveries at primary and secondary health facilities in Bangladesh: a case-observation study. PloS one. 2018;13(8):e0202508. 10.1371/journal.pone.0202508 PMC6095597 30114288

[48] Islam MA, Mamun A, Hossain MM, Bharati P, Saw A, Lestrel PE (2019). Prevalence and factors associated with early initiation of breastfeeding among Bangladeshi mothers: a nationwide cross-sectional study. PLoS One.

[49] Hobbs AJ, Mannion CA, McDonald SW, Brockway M, Tough SC (2016). The impact of caesarean section on breastfeeding initiation, duration and difficulties in the first four months postpartum. BMC Pregnancy Childbirth.

[50] Hung KJ, Berg O. Early skin-to-skin after cesarean to improve breastfeeding. MCN Am J Matern Child Nurs. 2011;36(5):318–24. 10.1097/NMC.0b013e3182266314 21743355

[51] Crenshaw JT, Cadwell K, Brimdyr K, Widström AM, Svensson K, Champion JD, Gilder RE, Winslow EH (2012). Use of a video-ethnographic intervention (PRECESS Immersion Method) to improve skin-to-skin care and breastfeeding rates. Breastfeed Med..

[52] Nolan A, Lawrence C (2009). A pilot study of a nursing intervention protocol to minimize maternal-infant separation after cesarean birth. J Obstet Gynecol Neonatal Nurs.

[53] Gouchon S, Gregori D, Picotto A, Patrucco G, Nangeroni M, Di Giulio P (2010). Skin-to-skin contact after cesarean delivery: an experimental study. Nurs Res..

[54] Haider MR, Rahman MM, Moinuddin M, Rahman AE, Ahmed S, Khan MM (2018). Ever-increasing caesarean section and its economic burden in Bangladesh. PLoS One.

[55] Betrán AP, Temmerman M, Kingdon C, Mohiddin A, Opiyo N, Torloni MR (2018). Interventions to reduce unnecessary caesarean sections in healthy women and babies. Lancet.

[56] Begum T, Nababan H, Rahman A, Islam MR, Adams A, Anwar I (2019). Monitoring caesarean births using the Robson ten group classification system: a cross-sectional survey of private for-profit facilities in urban Bangladesh. PLoS One.

[57] Begum T, Rahman A, Nababan H, Hoque DME, Khan AF, Ali T (2017). Indications and determinants of caesarean section delivery: evidence from a population-based study in Matlab, Bangladesh. PLoS One.

[58] Shahabuddin ASM, Delvaux T, Utz B, Bardají A, De Brouwere V (2016). Determinants and trends in health facility-based deliveries and caesarean sections among married adolescent girls in Bangladesh. BMJ Open.

[59] Kamal SM (2013). Preference for institutional delivery and caesarean sections in Bangladesh. J Health Popul Nutr.

[60] Rahman M (2009). Deliveries among adolescent mothers in rural Bangladesh: who provides assistance?. World Health Popul.

[61] Anwar I, Sami M, Akhtar N, Chowdhury ME, Salma U, Rahman M (2008). Inequity in maternal health-care services: evidence from home-based skilled-birth-attendant programmes in Bangladesh. Bull World Health Organ.

[62] Blanc AK, Warren C, McCarthy KJ, Kimani J, Ndwiga C, RamaRao S (2016). Assessing the validity of indicators of the quality of maternal and newborn health care in Kenya. J Glob Health.

[63] Stanton CK, Rawlins B, Drake M, Dos Anjos M, Cantor D, Chongo L (2013). Measuring coverage in MNCH: testing the validity of women's self-report of key maternal and newborn health interventions during the peripartum period in Mozambique. PLoS One.

[64] Safari K, Saeed AA, Hasan SS, Moghaddam-Banaem L (2018). The effect of mother and newborn early skin-to-skin contact on initiation of breastfeeding, newborn temperature and duration of third stage of labor. Int Breastfeed J.

